# Public Health Implications of High Cytomegalovirus Seroprevalence in Pregnancy: A Cross‐Sectional Study in Western Uganda

**DOI:** 10.1002/puh2.70258

**Published:** 2026-05-05

**Authors:** Bashir Mohamed Naima, Marie Pascaline Sabine Ishimwe, Theodore Nteziyaremye, Jean de Dieu Rukamba, Musa Kasujja, Theoneste Hakizimana

**Affiliations:** ^1^ Department of Obstetrics and Gynecology Kampala International University Ishaka Uganda; ^2^ Department of Pediatrics and Child Health Kampala International University Ishaka Uganda; ^3^ Department of Sciences University of Rwanda Kigali Rwanda

**Keywords:** cytomegalovirus infections, postpartum period, risk factors, seroprevalence, Uganda

## Abstract

**Background:**

Cytomegalovirus (CMV) is a common maternal infection associated with adverse fetal and neonatal outcomes, yet data among pregnant women in Uganda remain limited. This study assessed CMV seroprevalence and associated factors in western Uganda.

**Methods:**

We conducted a cross‐sectional study from January to April 2024 among 351 immediate postpartum women consecutively enrolled at Fort Portal Regional Referral Hospital in western Uganda, enrolled through consecutive sampling. Sociodemographic, obstetric, and medical data were collected using pretested questionnaires. Maternal serum samples were tested for CMV immunoglobulin G (IgG) and immunoglobulin M (IgM) using laboratory immunoassays (ELISA/CLIA). IgM‐positive or equivocal samples underwent reflex IgG avidity testing to differentiate recent primary from chronic or non‐primary infection. CMV serostatus was classified as seronegative or seropositive, with seropositive cases further categorized as acute or chronic infection. Data were analyzed using STATA version 14.2, applying bivariate and multivariate logistic regression at a 95% confidence level.

**Results:**

The overall CMV seroprevalence was 81.2% (95% confidence interval [CI]: 76.7%–84.97%). Chronic or non‐primary infection accounted for 78.9% of cases, whereas acute infection was identified in 2.3% of participants. CMV seropositivity was independently associated with maternal age 25–34 years (adjusted odds ratio [aOR]: 2.9, 95% CI: 1.44–5.83) and ≥35 years (aOR: 4.6, 95% CI: 1.80–11.52), rural residence (aOR: 2.2, 95% CI: 1.20–4.15), lower education levels, and a history of spontaneous abortion (aOR: 3.2, 95% CI: 1.21–8.72).

**Conclusions:**

CMV infection is highly prevalent among pregnant women in western Uganda, predominantly reflecting chronic infection. The identified sociodemographic and obstetric risk factors underscore the need for targeted public health education, consideration of antenatal CMV screening strategies, and interventions to reduce CMV‐related maternal and neonatal morbidity.

## Graphical abstract

Among immediate postpartum women in western Uganda, maternal CMV seroprevalence was high (81.2%) and was predominantly chronic or non‐primary infection. Older age, rural residence, lower education, and prior spontaneous abortion were associated with seropositivity, supporting targeted maternal CMV education and evaluation of context‐appropriate screening strategies.

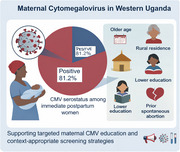

## 1 Introduction

Cytomegalovirus (CMV) is an enveloped DNA virus that is a member of the herpes family and belongs to a group of vertically transmitted infections known as TORCH [1]. The infection is usually asymptomatic in immunocompetent humans. However, during pregnancy, the primary infection or reactivation of latent infection can be transmitted vertically to the fetus and cause congenital CMV syndromes that include microcephaly, jaundice, prematurity, and intrauterine growth restriction [2, 3]. CMV can be transmitted horizontally through contact with infectious bodily fluids such as blood, saliva, urine, tears, seminal fluid, cervical secretions, and breast milk [4]. The serological diagnosis of maternal CMV is based on the detection of antibodies to CMV. Immunoglobulin G (IgG) antibodies indicate latent CMV infection, whereas immunoglobulin M (IgM) antibodies may indicate primary infections or reactivation of chronic infection [5, 6].

Globally, the CMV seroprevalence is estimated at 83% in the general population, with slightly higher rates of 86% among women of reproductive age and blood donors [7]. The prevalence of congenital CMV infections varies significantly, with the highest rates found in Africa at 3.71% and the lowest in the Eastern Mediterranean region at 0.53% [8]. A cross‐sectional study in China reported a seroprevalence of 39.2%, indicating substantial regional variations in infection rates [9].

In Africa, the overall pooled prevalence of latent CMV is reported to be 79% [10]. Specific studies have revealed high seroprevalence rates among pregnant women, such as 86.6% in Angola, with 84.6% showing latent infections and 2% active infections [11]. In Nigeria, the CMV seroprevalence is 87.9% for IgG and 7.8% for IgM among pregnant women, indicating a considerable proportion of recent infections [1].

CMV during pregnancy can be transmitted vertically to the fetus to cause congenital CMV, and it has been shown to be associated with early adverse neonatal outcomes, which include low APGAR score, stillbirth, death at the time of delivery, admission to the neonatal intensive care unit, low birth weight, hepatosplenomegaly, seizures, microcephaly, and jaundice [8, 12, 13, 14].

Individuals with less education are less likely to have access to information about disease prevention and hygiene practices, which influences awareness and behavior related to health maintenance, thereby reducing the likelihood of CMV transmission [15]. A systematic review by Fowler et al. on global prevalence revealed that lower education levels were significantly associated with higher CMV seroprevalence, underscoring the importance of health education [4]. Despite the studies done in Africa, they emphasized the seroprevalence of CMV during pregnancy, ignoring its associated factors.

In Uganda, CMV data remain limited and are drawn from only a few settings. A study at Kawempe National Referral Hospital in Kampala reported universal maternal CMV IgG seropositivity and 5.8% CMV IgM seropositivity among women admitted in labor [16]. In a multicenter Ugandan study, cord blood CMV positivity among newborn–mother pairs was 3%, and maternal vaginal shedding at parturition was 33% [8]. A recent community‐based study in eastern Uganda further reported congenital CMV infection in 0.4% of newborns [17]. These findings indicate substantial CMV exposure in Uganda but limited evidence from western Uganda, particularly on maternal seroprevalence and associated factors among women assessed around delivery. This study determined the seroprevalence of CMV infection and associated factors among immediate postpartum women at Fort Portal Regional Referral Hospital (FRRH) in western Uganda.

## 2 Materials and Methods

### 2.1 Study Design and Setting

This was a cross‐sectional study conducted from postnatal ward in the Department of Obstetrics and Gynecology of FRRH. FRRH is located in the Kabarole district of Fort Portal municipality in western Uganda. It is approximately 300 km from Kampala, the capital city of Uganda. The facility serves patients from the districts of Kabarole, Bundibugyo, Kamwenge, Kasese, Kyegegwa, Ntoroko, and Kyenjojo. The hospital has a bed capacity of 384 for inpatients. Internal medicine, surgery, pediatrics, and gynecology comprise its four principal departments. It has a well‐equipped modern laboratory that performs hematological, biochemical, microbiological, parasitological, and serological assays. Obstetrics and gynecology department is divided into six sectors, which include the gynecology outpatient clinic, antenatal care (ANC) clinic, gynecology ward, postnatal ward, antenatal ward, and labor suite. On average, the facility performs 15–20 deliveries per day.

### 2.2 Eligibility Criteria

#### 2.2.1 Inclusion Criteria

All mothers who delivered at FRRH during the study period and who provided consent to take part in the study.

#### 2.2.2 Exclusion Criteria

Mothers delivered at less than 26 weeks of gestation.

### 2.3 Sample Size Calculation

The sample size was determined using the Daniel formula for estimation of a single population proportion:


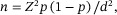

where *n* is the required sample size, *Z* is the standard normal deviate at the 95% confidence level (1.96), *p* is the estimated proportion from a previous related study, and *d* is the margin of error, set at 5%. Using *p* = 35.44% from a previous study by Zhang et al. [18] and *d* = 0.05, the calculated minimum sample size was


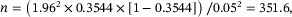

which was approximated to 352 participants. In this study, 351 immediate postpartum women were enrolled and analyzed.

### 2.4 Data Collection Instruments

A questionnaire was used to collect information from all study participants who consented to participate in the study. One part of the questionnaire gathered data on sociodemographic and clinical factors, whereas another part collected information from blood samples, including the types of CMV antibodies. The questionnaire was translated into Rutooro for participants who could not understand English.

### 2.5 Study Procedure

Immediate postpartum mothers (at least 12 h postdelivery) were assessed for eligibility criteria. Those who met the inclusion criteria were informed about the study, provided written consent, and completed the questionnaire. Those who did not meet inclusion criteria and those who did not consent underwent routine care. For CMV prevalence, blood samples were collected from every participant for serological testing. Routine care was given to all mothers and newborns according to Uganda's clinical guidelines, and the researcher followed up on the laboratory results. During the study period, all eligible immediate postpartum women present in the postnatal ward were approached consecutively for recruitment until the required sample size was attained. A total of 370 women were screened, 351 consented and were enrolled, and 19 declined participation. No additional data were collected from women who declined consent, in accordance with ethical requirements.

### 2.6 CMV Testing Procedure

After informed consent and completion of a pretested questionnaire, 3–5 mL of maternal venous blood was collected into a serum‐separator tube, allowed to clot for 30–60 min, and centrifuged at 1500–2000 *g* for 10 min. Serum CMV IgG and IgM were measured using the Elecsys CMV IgG assay and Elecsys CMV IgM assay (Roche Diagnostics, Mannheim, Germany) on a cobas e 601 analyzer, according to the manufacturer's instructions. For the CMV IgG assay, results were interpreted as nonreactive at <0.5 U/mL, borderline at 0.5–<1.0 U/mL, and reactive at ≥1.0 U/mL. For the CMV IgM assay, results were interpreted as nonreactive at <0.7 cutoff index (COI), indeterminate at 0.7–<1.0 COI, and reactive at ≥1.0 COI. Reported assay performance in a recent Ugandan pregnancy study using this platform was 96.6% specificity and 100% sensitivity for CMV IgG and 92.3% specificity and 93.9% sensitivity for CMV IgM. Samples with positive or equivocal CMV IgM results were reflexed for CMV IgG avidity testing using the Elecsys CMV IgG Avidity assay (Roche Diagnostics) on the same serum aliquot at a collaborating, accredited laboratory. Avidity was interpreted according to the manufacturer's thresholds as low avidity (<45.0%), gray‐zone avidity (45.0%–54.9%), and high avidity (≥55.0%). Maternal CMV serostatus was classified as seronegative (IgG−/IgM−) or seropositive. Seropositive women were further subclassified as having acute infection if they were IgM positive with low IgG avidity, or chronic/non‐primary infection if they were IgG positive with IgM negative results or IgM positive with high IgG avidity. Each analytical run included internal positive and negative controls, and 5%–10% of samples were retested for quality assurance. Pre‐analytic conditions, including time to centrifugation and storage at 2–8°C on‐site and −20°C/−80°C for batched referral, were documented throughout the testing process.

### 2.7 Study Variables

The study assessed various independent variables to understand their associations with CMV infection among women. The independent variables were sociodemographic, obstetrical, and medical factors, which included age, marital status, education, occupation, residence, parity, history of abortions, history of intrauterine fetal death (IUFD), history of human immune virus (HIV) infection, and history of blood transfusion during pregnancy. The dependent variable was CMV infection as a binary variable confirmed through laboratory testing. HIV status was obtained from the participant's ANC card based on documented routine antenatal HIV test results and was analyzed as a maternal medical factor. Women delivering before 26 completed weeks of gestation were excluded because periviable deliveries often require urgent maternal and neonatal stabilization, which may make standardized postpartum interview and blood collection difficult within the study window.

### 2.8 Data Quality Control

The research assistants were sufficiently educated and continually monitored by the lead investigator to guarantee that the right data were acquired. The inclusion and exclusion criteria were rigorously adhered to. Each questionnaire was reviewed for completeness before and after data collection. The ethical requirements were rigorously adhered to, and blood samples were obtained under sterile environment. Serological testing of CMV and interpretation of findings were done following standard procedures.

### 2.9 Data Analysis

The principal investigator checked all questionnaires for completeness and accuracy before data entry. Data were entered into Microsoft Excel 2016, double‐entered, and validated to minimize inconsistencies, then exported to STATA version 14.2 for analysis. Multiple backup copies of the dataset were stored securely in different locations.

The seroprevalence of CMV was calculated as the proportion of women who tested positive for anti‐CMV antibodies among all women tested and was summarized using frequencies and percentages. The results were presented in a pie chart.

To assess factors associated with CMV seropositivity, bivariable and multivariable logistic regression analyses were performed. Sociodemographic, obstetric, and medical characteristics were treated as independent variables, whereas CMV serostatus was treated as the dependent binary outcome. Unadjusted odds ratios, 95% confidence intervals (CIs), and *p* values were reported at bivariable analysis. Variables with a *p* value of ≤0.20 were considered for inclusion in the multivariable logistic regression model. Adjusted odds ratios (aORs) with 95% CIs were then reported for the final model, and statistical significance was set at *p* ≤ 0.05.

Data were checked for completeness, accuracy, and consistency before analysis. No missing data were identified for the variables included in the regression analyses; therefore, complete‐case analysis was performed. Multicollinearity among candidate independent variables was assessed using variance inflation factors, and no significant multicollinearity was identified. Model fit for the final multivariable logistic regression model was evaluated using the Hosmer–Lemeshow goodness‐of‐fit test and pseudo‐*R* ^2^, and the final model demonstrated acceptable fit to the data.

## 3 Results

### 3.1 Descriptive Characteristics of the Study Participants

**FIGURE 1:**
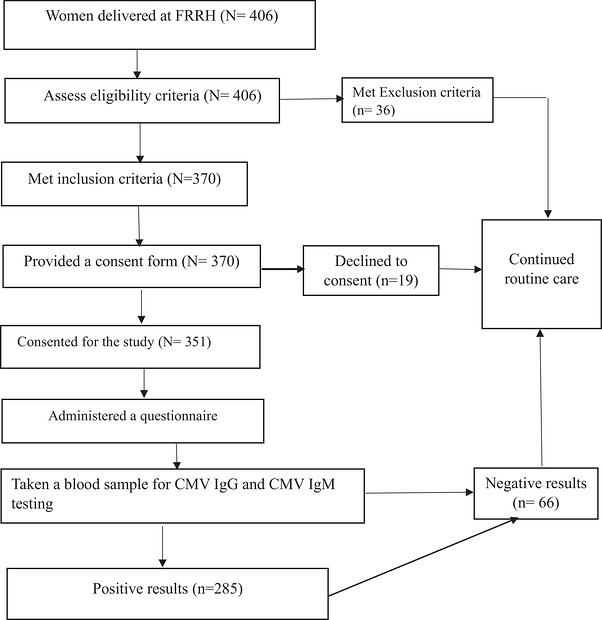
Participant flow diagram showing screening, exclusions, consent, enrolment, and analysis of immediate postpartum women at Fort Portal Regional Referral Hospital. CMV, cytomegalovirus; FRRH, Fort Portal Regional Referral Hospital; IgG, immunoglobulin G; IgM, immunoglobulin M.

A total of 406 immediate postpartum women were screened during the study period, of whom 36 met exclusion criteria, whereas 19 declined participation. Finally, 351 consented and were enrolled in the study (Figure 1). Among 351 respondents considered in this study, majority were aged 25–34 (58.69%), resided in rural areas (71.23%), were married (86.32%), and had secondary education (57.26%). The majority had a monthly income of 500,000 Ugandan shillings or less (92.02%) and were unemployed (53.56%). The obstetric histories showed that most participants were multiparous (66.38%), with some having a history of spontaneous abortion (20.51%) or IUFD (12.25%). Additionally, 11.97% were HIV positive, and 14.25% had a history of blood transfusion (Table 1).

**TABLE 1:** Sociodemographic, obstetrics, and medical characteristics of the study participants (*N* = 351).

Variable	Category	Frequency (*n*)	Percentages
**Sociodemographic characteristics**			
Age	15–24	62	17.66
	25–34	206	**58.69**
	≥35	83	23.65
Residence	Urban	101	28.77
	Rural	250	**71.23**
Marital status	Unmarried	48	13.68
	Married	303	**86.32**
Level of education	No formal	19	5.41
	Primary or lower	103	29.34
	Secondary	201	**57.26**
	Tertiary	28	7.98
Monthly income	≤500,000/‐	323	**92.02**
	>500,000/‐	28	7.98
Employment status	Formal	49	13.96
	Informal	114	32.48
	Unemployed	188	**53.56**
**Obstetrics characteristics**			
Parity	Nullipara	73	20.80
	Primipara	45	12.82
	Multipara	233	**66.38**
History of spontaneous abortion	No	279	**79.49**
	Yes	72	20.51
History of IUFD	No	308	**87.75**
	Yes	43	12.25
**Medical characteristics**			
HIV status	Negative	309	**88.03**
	Positive	42	11.97
Previous history of blood transfusion	No	301	**85.75**
	Yes	50	14.25

Abbreviations: HIV, human immune virus; IUFD, intrauterine fetal death.

Bold values indicate maority of participants in a give category of basic characteristics of participants.

### 3.2 The Seroprevalence of CMV Infection Among Mothers Who Delivered at FRRH

**FIGURE 2:**
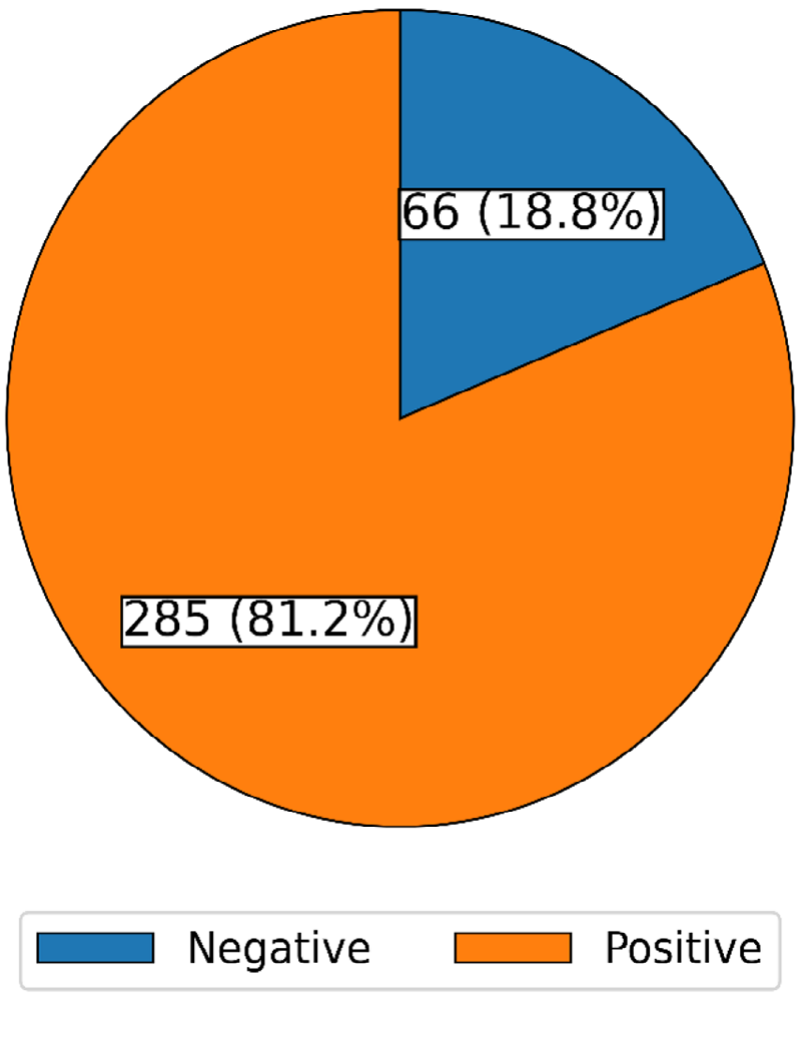
Pie chart showing the seroprevalence of CMV infection among mothers who delivered at Fort Portal Regional Referral Hospital.

Out of 351 pregnant women tested, 285 were positive for anti‐CMV antibodies, with a seroprevalence of 81.2% (95% CI: 76.7%–84.97%). Conversely, 66 women tested negative, yielding a seronegativity rate of 18.8% (95% CI: 15.0%–23.3%) (Figure 2). The proportion of chronic CMV cases was 277/351 (78.9%), and that of acute CMV cases was 8/351 (2.3%).

### 3.3 Factors Associated With CMV Infection Among Mothers Delivered at FRRH

At bivariable level of analysis, variables considered for multivariate analysis were age, residence, level of education, parity, history of spontaneous abortion, and history of IUFD (Table 2). After removing confounders, the multivariable analysis revealed that women aged 25–34 years are nearly 3 times more likely, and those aged 35 years and above are more than 4.5 times more likely to be seropositive for anti‐CMV antibodies compared to those aged 15–24 years. Women residing in rural areas are nearly 2.2 times more likely to be seropositive than those in urban areas. In terms of education, women with primary education are 3.3 times more likely, and those with secondary education are nearly 3 times more likely to be seropositive than those with tertiary education. Additionally, women with a history of spontaneous abortion are 3.2 times more likely to be seropositive than those without such a history (Table 3).

**TABLE 2:** Bivariate analysis of sociodemographic, obstetric, and medical factors associated with cytomegalovirus among mothers delivered at Fort Portal Regional Referral Hospital (*N* = 351).

		Seroprevalence of anti‐CMV			
Variable	Category	Negative (*n* = 66) (%)	Positive (*n* = 285) (%)	cOR (95% CI)	*p* value
Age	15–24	24 (38.71)	38 (61.29)	1	
	25–34	33 (16.02)	173 (83.98)	3.3 (1.759–6.231)	0.023*
	≥35	9 (10.84)	74 (89.16)	5.2 (2.197–12.274)	0.001*
Residence	Urban	26 (25.74)	75 (74.26)	1	
	Rural	40 (16.00)	210 (84.00)	1.8 (1.040–3.185)	0.036*
Marital status	Married	56 (18.48)	247 (81.52)	1	
	Unmarried	10 (20.83)	38 (79.17)	0.9 (0.405–1.832)	0.699
Level of education	No formal	2 (10.53)	17 (89.47)	1.8 (0.508–6.464)	0.36
	Primary	18 (17.48)	85 (82.52)	3.8 (1.490–9.673)	0.005*
	Secondary	35 (17.41)	166 (82.59)	3.1 (1.323–7.119)	0.009*
	Tertiary	11 (39.29)	17 (60.71)	1	
Monthly income	>500,000/‐	6 (21.43)	22 (78.57)	1	
	≤500,000/‐	60 (18.58)	263 (81.42)	1.2 (0.465–3.076)	0.711
Employment	Formal	10 (20.41)	39 (79.59)	1	
	Informal	23 (20.18)	91 (79.82)	1.0 (0.429–2.264)	0.973
	Unemployed	33 (17.55)	155 (82.45)	1.2 (0.657–2.146)	0.57
Parity	Nullipara (P0)	21 (28.77)	52 (71.23)	1	
	Primipara (P1)	7 (15.56)	38 (84.44)	2.2 (0.850–5.681)	0.106*
	Multipara (P2+)	38 (16.31)	195 (83.69)	2.1 (1.121–3.831)	0.020*
History of spontaneous abortion	No	61 (21.86)	218 (78.14)	1	
	Yes	5 (6.94)	67 (93.06)	3.7 (1.447–9.714)	0.007*
History of IUFD	No	63 (20.45)	245 (79.55)	1	
	Yes	3 (6.98)	40 (93.02)	3.4 (1.027–11.446)	0.045*
HIV	No	61 (19.74)	248 (80.26)	1	
	Yes	5 (11.90)	37 (88.10)	1.8 (0.687–4.825)	0.229
Previous history of BT	No	58 (19.27)	243 (80.73)	1	
	Yes	8 (16.00)	42 (84.00)	1.3 (0.558–2.813)	0.584

Abbreviations: BT, blood transfusion; CI, confidence interval; CMV, cytomegalovirus; cOR, crude odds ratio; IUFD, intrauterine fetal death; UGX, Uganda shillings.

* *p* ≤ 0.2.

**TABLE 3:** Factors associated with cytomegalovirus infection among mothers delivered at Fort Portal Regional Referral Hospital.

Variable	Category	cOR (95% CI)	*p* value	aOR (95% CI)	*p* value
Age	15–24	Ref.		Ref.	
	25–34	3.3 (1.759–6.231)	0.023^*^	2.9 (1.436–5.828)	0.003^**^
	≥35	5.2 (2.197–12.274)	0.001^*^	4.6 (1.800–11.524)	0.001^**^
Residence	Urban	Ref.		Ref.	
	Rural	1.8 (1.040–3.185)	0.036^*^	2.2 (1.203–4.152)	0.011^**^
Level of education	No formal	1.8 (0.508–6.464)	0.360	4.7 (0.783–28.4176)	0.091
	Primary	3.8 (1.490–9.673)	0.005^*^	3.3 (1.098–8.710)	0.033^**^
	Secondary	3.1 (1.323–7.119)	0.009*	2.9 (1.109–7.520)	0.030^**^
	Tertiary	Ref.		Ref.	
Parity	Nullipara (P0)	Ref.		Ref.	
	Primipara (P1)	2.2 (0.850–5.681)	0.106^*^	1.5 (0.499–4.425)	0.476
	Multipara (P2+)	2.1 (1.121–3.831)	0.020^*^	1.6 (0.780–3.100)	0.210
History of spontaneous abortion	No	Ref.		Ref.	
	Yes	3.7 (1.447–9.714)	0.007^*^	3.2 (1.208–8.723)	0.020^**^
History of IUFD	No	Ref.		Ref.	
	Yes	3.4 (1.027–11.446)	0.045^*^	2.7 (0.764–9.308)	0.124

Abbreviations: aOR, adjusted odds ratio; CI, confidence interval; cOR, crude odds ratio; IUFD, intrauterine fetal death.

* *p* ≤ 0.2.

** *p* ≤ 0.05.

CMV seropositivity was higher among women aged 25–34 years and ≥35 years than among those aged 15–24 years, higher among rural than urban residents, and higher among women with lower educational attainment than among those with tertiary education.

## 4 Discussion

This study found a high CMV seroprevalence of 81.2% among immediate postpartum women at FRRH, with most seropositive women showing chronic or non‐primary infection. Our findings should be interpreted primarily against studies conducted among pregnant or postpartum women rather than against highly selected populations such as blood donors, transplant recipients, or HIV‐only cohorts. In Uganda, a recent study at Kawempe National Referral Hospital reported universal CMV IgG seropositivity and 5.8% CMV IgM seropositivity among women admitted in labor [16]. Ugandan infant studies have also shown ongoing CMV burden, including 3% cord‐blood CMV positivity and 33% maternal vaginal shedding at parturition in newborn–mother pairs, as well as a 0.4% prevalence of congenital CMV in eastern Uganda [8, 17]. Comparable studies in the region similarly report high maternal CMV exposure, including CMV IgG seroprevalence of 73.9% in Mwanza, Tanzania, and 88.7% in southern Ethiopia [19, 20]. Differences across studies likely reflect variation in study populations, timing of sampling, laboratory methods, and background endemic exposure.

Older maternal age was independently associated with CMV seropositivity in our study. This is biologically plausible because CMV is a lifelong latent infection, and increasing age reflects greater cumulative opportunity for exposure before and during the reproductive years. A similar age‐related increase in CMV seropositivity has been reported in pregnant women in Tanzania, where the likelihood of CMV IgG seropositivity increased with age [19]. In addition, Fowler et al. reported in their global systematic review that CMV seroprevalence among women of reproductive age correlates with age and other markers of social disadvantage [4]. These findings suggest that the age association in our study is more likely to reflect cumulative lifetime exposure than a pregnancy‐specific biological effect.

Women residing in rural areas and those with lower educational attainment were also more likely to be CMV seropositive. These associations may reflect social and structural determinants of infection, including differences in hygiene opportunities, crowding, health literacy, and access to healthcare and preventive information. In western Romania, Gorun et al. likewise found higher CMV seroprevalence among women from rural areas than among those from urban areas [21]. Fowler et al. further reported that CMV seroprevalence correlates with socioeconomic status and education level globally [4]. In our setting, the observed effects of rural residence and lower education may therefore indicate broader patterns of social vulnerability rather than isolated demographic associations.

A history of spontaneous abortion was independently associated with CMV seropositivity in our analysis. However, this finding should be interpreted cautiously. Because this was a cross‐sectional study, temporal and causal relationships cannot be established. The observed association may reflect prior CMV exposure, residual confounding, or shared risk factors rather than a direct causal effect of CMV on previous pregnancy loss. Similar associations between CMV seropositivity and poor obstetric history have been reported in Tanzania and Iraq [19, 22], but these findings should be interpreted as hypothesis‐generating rather than confirmatory evidence that CMV caused prior spontaneous abortion.

An important clinical implication of our findings is that 18.8% of women remained seronegative at delivery and may, therefore, still be at risk of primary CMV acquisition in a future pregnancy. This subgroup may benefit from preconception or antenatal counseling on hygiene measures that reduce exposure to saliva and urine from young children. At the same time, the 2.3% of women classified as having acute infection highlights the need for further research on targeted maternal testing strategies in high‐burden settings. Nevertheless, routine universal maternal serologic screening is not currently recommended by major professional guidance. If neonatal testing is considered, congenital CMV is best diagnosed using PCR on saliva with confirmatory urine testing, and samples should be obtained within the first 21 days of life to distinguish congenital from postnatal infection [23, 24].

Taken together, the associations with older age, rural residence, lower education, and previous spontaneous abortion suggest that CMV exposure in this setting reflects both cumulative lifetime exposure and social vulnerability. The predominance of chronic or non‐primary infection further indicates that most women had been exposed before or during earlier reproductive years. These findings support strengthening maternal CMV education and justify further evaluation of context‐appropriate targeted maternal and newborn CMV screening approaches in Uganda, rather than immediate universal implementation.

Although there is increasing international advocacy for first‐trimester maternal CMV screening in pregnancy, routine universal maternal serologic screening is not currently recommended by major professional guidance. Therefore, in our setting, these findings support further evaluation of targeted maternal or newborn CMV screening approaches rather than immediate universal implementation.

### 4.1 Study Strengths and Limitations

The use of well‐established serological testing methods, including CMV IgG and IgM detection by laboratory immunoassay (ELISA/CLIA), enhanced the accuracy and reliability of the seroprevalence estimates. In addition, the application of standardized laboratory procedures strengthened the internal validity of the study findings.

However, the cross‐sectional design represents an important limitation, as it precludes the establishment of temporal or causal relationships between CMV seroprevalence and associated factors such as maternal age, residence, education level, and history of spontaneous abortion. Although this design was appropriate for estimating CMV seroprevalence and identifying associated factors, longitudinal or cohort studies following women from early pregnancy through delivery are needed to better characterize the timing of CMV acquisition and its impact on maternal and neonatal outcomes.

Because participants were recruited consecutively from a single regional referral hospital and rural residents constituted most of the sample, the findings may overrepresent referral‐dependent and rural populations and may not be fully generalizable to all pregnant or postpartum women in western Uganda.

## 5 Conclusions

This study found a high seroprevalence of maternal CMV infection among immediate postpartum women at FRRH in western Uganda, with most seropositive women showing chronic or non‐primary infection. Older maternal age, rural residence, lower education level, and a history of spontaneous abortion were independently associated with CMV seropositivity. In addition, nearly one in five women remained seronegative at delivery and may therefore be at risk of primary CMV infection in a future pregnancy.

### 5.1 Public Health Implications and Recommendations

These findings support strengthening maternal CMV health education, with particular emphasis on targeted counseling about hygiene and infection‐prevention measures among women of reproductive age, especially those in higher risk social and geographic groups. Furthermore, although there is growing discussion and increasing support in some recent recommendations for maternal CMV screening during pregnancy, routine universal maternal serologic screening is not yet recommended by major professional guidance. Given the absence of local feasibility, affordability, and cost‐effectiveness data, our findings should therefore be interpreted as supporting further evaluation of targeted antenatal or newborn CMV screening strategies in Uganda rather than immediate universal implementation. Future studies should assess the feasibility of first‐trimester maternal testing in selected higher risk populations, linkage to confirmatory IgG avidity testing where indicated, and pilot newborn screening using saliva or urine PCR within the first 21 days of life in referral settings.

## Author Contributions

Bashir Mohamed Naima developed the proposal and participated in data collection and data analysis. Theoneste Hakizimana and Marie Pascaline Sabine Ishimwe contributed significantly to the data collection, data entry, analysis, and drafting of the manuscript. Musa Kasujja and Jean de Dieu Rukamba participated in making corrections to the proposal and analysis process. Musa Kasujja, Theodore Nteziyaremye, and Marie Pascaline Sabine Ishimwe contributed to the revision of manuscript. All authors have read and approved the final manuscript.

## Funding

The authors have nothing to report.

## Ethics Statement

Ethical approval for the study was obtained from the Research and Ethics Committee of Bishop Stuart University (BSU‐REC‐2023‐248). Written informed consent was obtained from adult participants before enrolment. For participants younger than 18 years, written assent from the adolescent participant and written permission from a parent or legally authorized guardian were obtained, in accordance with the approved ethics protocol.

## Consent

The authors have nothing to report.

## Conflicts of Interest

The authors declare no conflicts of interest.

## Data Availability

The datasets utilized in this study can be obtained from the corresponding author upon request. Please contact Dr. Theoneste Hakizimana via email at theonestehakizmana5@gmail.com.
